# Psychometric Properties of the Spanish Version of the Caregiver Contribution to Self-Care of Diabetes Inventory (CC-SCODI)

**DOI:** 10.3390/nursrep15040129

**Published:** 2025-04-12

**Authors:** Jesús Martínez-Tofé, Iván Santolalla-Arnedo, Vicente Gea-Caballero, Angela Durante, Antonio Martínez-Sabater, Mercedes Sánchez-Barba, Marco Di Nitto, Pilar Sanchez-Conde, Silvia Gónzalez-Fernández, Regina Ruíz de Viñaspre-Hernández, Raúl Juárez-Vela, Nelia Soto-Ruiz

**Affiliations:** 1 Doctoral Program in Health Sciences, Public University of Navarra, 31008 Pamplona, Spain; jesus.martinezt@unirioja.es; 2 GRUPAC Research Group in Care, Faculty of Health Sciences, University of La Rioja, 26004 Logroño, Spain; ivan.santolalla@unirioja.es (I.S.-A.) reruizde@unirioja.es (R.R.d.V.-H.); 3 Faculty of Health Sciences, International University of Valencia, 46002 Valencia, Spain; vagea@universidadviu.com; 4 Health Science Interdisciplinary Center, Sant’Anna School of Advanced Studies, 56127 Pisa, Italy; angela.durante@santannapisa.it; 5 Fondazione Toscana Gabriele Monasterio, 54100 Pisa, Italy; 6 Faculty of Health Sciences, University of Valencia, 46010 Valencia, Spain; antonio.martinez-sabater@uv.es; 7 Nursing Care and Education Research Group (GRIECE), GIUV2019-456, Nursing Department, Universitat de Valencia, 46010 Valencia, Spain; 8 Care Research Group (INCLIVA), Hospital Clínico Universitario de Valencia, 46010 Valencia, Spain; 9 Department of Statistics, Faculty of Medicine, University of Salamanca, 37007 Salamanca, Spain; 10 Department of Health Sciences, University of Genoa, Via A. Pastore 1, 16132 Genoa, Italy; marco.dinitto@unige.it; 11 University Hospital of Salamanca, 37007 Salamanca, Spain; pconde@usal.es; 12 Faculty of Medicine, University of Salamanca, 37007 Salamanca, Spain; sigofe@usal.es; 13 Department of Health Sciences, Public University of Navarra, 31008 Pamplona, Spain; nelia.soto@unavarra.es; 14 IdiSNA—Navarra Institute for Health Research, 31008 Pamplona, Spain

**Keywords:** caregiver contribution, diabetes mellitus, inventory, self-care, Spain, validation, nursing

## Abstract

**Background:** The Caregiver Contribution to Self-Care of Diabetes Inventory (CC-SCODI) is an instrument grounded in the middle-range theory of self-care of chronic illness. It is designed to measure how caregivers support individuals with diabetes mellitus in carrying out self-care activities. Effective tools are essential for clinicians and researchers to evaluate factors influencing self-care, including caregivers’ contributions. This study aimed to assess the reliability and validity of the Spanish translation of the CC-SCODI. **Methods:** A total of 201 caregivers of individuals with Type 1 Diabetes Mellitus (T1DM) and Type 2 Diabetes Mellitus (T2DM) were recruited for participation in this cross-sectional study. Participants were selected through convenience sampling at a university hospital. Before administration, the survey questions were translated and culturally adapted to ensure appropriateness for both patients and caregivers. Confirmatory factor analysis (CFA) was performed on each of the CC-SCODI subscales using models fitted to the relevant indices. **Results:** The initial construction of the four-dimensional tool was verified. The internal consistency of the four subscales was assessed using Cronbach’s α to measure the caregiver contribution to patients’ self-care maintenance (α = 0.725), self-care monitoring (α = 0.728), self-care management (α = 0.729), and caregiver self-efficacy in contributing to patient self-care (α = 0.921). Model fit indices demonstrated a chi-square value of 1.028 with 773 degrees of freedom. CFA indicated an excellent model fit, confirming the reliability and validity of the proposed structure. **Conclusions:** The internal consistency and reliability of the Spanish version of the CC-SCODI were deemed adequate. Due to its strong psychometric properties, this instrument is considered appropriate for evaluating the contribution of caregivers to the self-care behaviors of Spanish-speaking individuals with diabetes.

## 1. Introduction

One of the biggest public health issues in the world is diabetes mellitus (DM) [1]. It is linked to a lower quality of life, higher morbidity, and a shorter life expectancy [2,3]. Among the complications of DM are myocardial infarction, stroke, renal failure, blindness, and amputations [4]. About 529 million individuals worldwide had DM in 2021, and by 2050, that number is predicted to rise to over 1310 million [5]. In Spain, where this study was conducted, the prevalence is the second highest in Europe, according to the International Diabetes Federation (IDF), reaching 14.8% of the population [6]. Chronic diseases, such as DM, decrease the quality of life of patients and their informal caregivers and increase healthcare costs [7]. Healthcare systems and providers might encourage patients to take care of themselves to alleviate this burden [8]. When developing self-care interventions, Jaarsma et al. [9] recommended applying the theory to establish a framework for planning and assessment. The middle-range theory of self-care of chronic illness posits that self-care is a naturalistic decision-making process aimed at maintaining stable health conditions through illness management and health promotion [8]. Appropriate self-care behaviors have been associated with reduced hospitalization and mortality rates, as well as improved patient quality of life and overall well-being [10]. Self-care places individuals at the center of disease management and is recognized as a fundamental component of diabetes mellitus (DM) therapy [11].

The treatment of DM has traditionally focused on the patient and health services [12]. However, people with diabetes spend only about 1% of their time with healthcare professionals. Therefore, patients themselves, together with their families, have to take control and management of their own treatment [13]. For this reason, families represent one of the most critical units in the care of chronic diseases within the community [14]. Research findings indicate that most patients with DM receive care at home from informal caregivers [15]. Informal caregivers are defined as family members or acquaintances who provide unpaid care to a patient with whom they have a personal relationship [16]. In addition to assisting with or performing routine self-care tasks, caregivers play a vital role in ensuring adherence to medical treatment recommendations and often share responsibilities in managing the illness [17].

Caregiver support has been shown to enhance self-management and improve the quality of life of patients with DM [18]. Conversely, a lack of informal caregiver support can negatively affect medication adherence and blood glucose control [19]. Within the family and affective environment, care provided by non-professionals is a cornerstone of the social support system. For instance, it is estimated that, in 2008, approximately 3249 million hours of informal care were provided to dependents aged 65 and older in Spain. The monetary value of these hours was estimated to range between EUR 24,918 and 41,291 million [20].

The term “caregiver contribution to self-care” (CC) refers to the process by which caregivers propose or substitute practices that promote the stability of chronic diseases and the management of their symptoms [21]. Evidence suggests that caregivers of individuals with diabetes mellitus (DM) have a positive impact on clinical, economic, and humanistic outcomes, including improved patient adherence to follow-up care and enhanced diabetes-related metrics [22]. However, the role of informal caregivers in supporting self-care among individuals with diabetes has received limited attention.

One of the primary reasons for this gap may be the lack of valid, reliable, and specific instruments to assess such contributions. While validated tools exist to measure general aspects of caregiving, such as the Caregiver Preparedness Scale [23], the Connor-Davidson Resilience Scale [24], or instruments assessing caregiver burden [25], these tools do not specifically address the unique contributions of caregivers to diabetes self-care. Additionally, instruments such as the Multidimensional Diabetes Questionnaire provide a diabetes-specific approach but focus solely on social support as the only factor related to caregiver contribution [26].

Over the past few decades, several tools have been developed to measure self-care in both general and specific chronic diseases, many grounded in Riegel’s “middle-range theory of self-care of chronic illness” [8]. Among these, the Self-Care Inventory for Diabetes (SCODI) developed by Ausili et al. in 2017 has been identified as a relevant and reliable tool for monitoring diabetes self-care, with multidimensional model-based reliability ranging from 0.81 (maintenance) to 0.89 (confidence). Statistically significant correlations were observed between self-care monitoring and diabetes complications (*p* = 0.04), as well as between self-care maintenance and HbA1c levels (*p* = 0.02), making it valuable for researchers and healthcare professionals [27].

The SCODI measures key elements of Riegel’s theory, including self-care maintenance, self-care monitoring, and self-care management, as well as self-care self-efficacy. Self-care maintenance behaviors include preserving physical and mental equilibrium, improving well-being through nutrition, exercise, or medication, and maintaining overall health. Self-care monitoring encompasses behaviors aimed at observing and interpreting bodily signals, such as blood glucose monitoring, which links self-care to maintaining one’s health. Self-care management refers to responding to cues, such as hypoglycemia or hyperglycemia, by taking actions like administering extra insulin. While self-care self-efficacy significantly influences self-care maintenance, monitoring, and management, it is not considered a direct component of self-care. Self-efficacy reflects perseverance in the face of challenges and self-assurance in one’s ability to engage in self-care [22,23,27].

The SCODI has demonstrated consistent dimensionality and high reliability across individuals with type 1 diabetes (T1DM) and type 2 diabetes (T2DM), with minimal variation in factor loadings for each scale and component [28].

In parallel, several tools have been developed to assess caregiver contribution (CC) to self-care for chronic illnesses. Many of these tools are conceptually aligned with those that measure patient self-care, following Riegel’s theory and the Caregiver Contribution to Self-Care Theory in Heart Failure [21].

The original Caregiver Contribution to Self-Care of Diabetes Inventory (CC-SCODI) scale, the caregiver version of the SCODI, was developed by Fabrizi and colleagues and tested in a multicenter cross-sectional study on an Italian sample of patients with type 2 diabetes [29]. The validation paper of the original scale is currently under review, and this is the first translated version of the CC-SCODI to undergo validation. While the SCODI asks patients to report the frequency of their self-care behaviors, the CC-SCODI asks caregivers to indicate how often they suggest or perform self-care behaviors on behalf of the patient. The CC-SCODI consists of 40 items and evaluates the same four dimensions as the SCODI: maintenance, monitoring, management, and self-efficacy. The SCODI and CC-SCODI are mirror instruments, as they assess the same behaviors but from different perspectives.

Some of the caregiver contribution instruments have already undergone psychometric and validation testing [30,31,32]. The CC-SCODI has been used in some studies [33] and is available in several languages at https://self-care-measures.com, and the original scale validation article is currently under review [29]. The Spanish version of the SCODI has been validated [34], but no validated translation of the CC-SCODI is available.

Thus, the objective of this study was to translate, adapt, and evaluate the psychometric properties of the Caregiver Contribution to Diabetes Self-Care Inventory (CC-SCODI) for use in Spanish-speaking populations.

## 2. Materials and Methods

### 2.1. Study Design

A single-center, cross-sectional study was conducted in northern Spain (Logroño) to evaluate/assess the psychometric properties of the CC-SCODI. The process of cultural adaptation and large-scale translation adhered to the methodology proposed by Beaton et al., which involves a rigorous, multi-step approach: initial translation, synthesis of translations, back-translation, retrosynthesis, evaluation by a professional committee, and pretesting [35]. This process ensures both linguistic and cultural equivalence of the instrument in the target language. Additionally, the guidelines provided by the original authors for the cultural adaptation and translation of the tool, as outlined on the Self-Care Measures website, were strictly followed throughout the entire procedure. Consequently, the Spanish translation was based on the English version of the CC-SCODI, developed by the same team that created the original version of the scale [29].

The initial translation from English to Spanish was independently performed by two expert translators who were native Spanish speakers. One translator was a professional linguist with no background in healthcare, ensuring a focus on linguistic accuracy, while the other was a healthcare professional (nurse) with substantial familiarity with the instrument and its conceptual framework. Following a collaborative session involving both translators and academic experts, the two independent translations were synthesized into a unified Spanish version.

Subsequently, the Spanish version underwent a back-translation process conducted by two professional translators whose first languages were English. These translators had no prior exposure to the original English version of the instrument or the concept of self-care, ensuring an unbiased and independent back-translation. The back-translated English version was compared with the original text to identify and resolve any discrepancies, further validating the accuracy and cultural relevance of the Spanish version.

The research group, in collaboration with the translation team, synthesized the two back-translated versions to produce a final English back-translation. This version was subsequently submitted to the original author of the instrument for review, approval, and verification of its accuracy in reflecting the content and intent of the original tool. To ensure the cultural and linguistic appropriateness of the final Spanish version, cognitive interviews were conducted with a sample of 30 respondents. These interviews facilitated the identification of potential ambiguities or misunderstandings in the translated items, allowing for minor adjustments to be made to the final edition of the instrument.

### 2.2. Data Analysis and Procedures

A single-center, cross-sectional study was conducted in northern Spain (Logroño, La Rioja) to evaluate the psychometric properties of the CC-SCODI.

#### 2.2.1. Sample Selection and Inclusion Criteria

According to several authors [36], an acceptable and reliable sample size for component analysis in confirming validity ranges from 5 to 10 participants per item. Although the CC-SCODI was designed as a four-part inventory, it does not provide a general indicator of self-care. Given that the self-care maintenance subscale contains the largest number of items (12), a minimum of 84 participants was deemed sufficient. However, to ensure a more stable analysis and alignment with the initial validation of the SCODI [27] and other similar instruments assessing caregiver contributions [30,31,32,37], the study aimed to recruit a minimum of 200 participants.

Convenience sampling was employed to invite all patient–caregiver dyads admitted to the multipurpose surgical ward of the Hospital Universitario San Pedro de Logroño between January 2022 and January 2023. Eligibility criteria for patients included a medical diagnosis of T1DM or T2DM, age ≥ 18 years, informed consent, and awareness of the study’s objectives. Patients were excluded if they had significant cognitive impairment (scoring < 4 points on a “six-item screener” cognitive assessment questionnaire [29]) or if they had been diagnosed with DM for less than one year. Caregivers were required to be the primary caregiver for an eligible patient, be ≥18 years old, sign an informed consent form, and understand the study’s objectives. Caregivers with significant cognitive impairment (scoring < 4 points on the same “six item screener” questionnaire [38]) were excluded.

#### 2.2.2. Data Collection

In addition to the Spanish version of the CC-SCODI, parallel sociodemographic questionnaires and inquiry forms were used to collect data on caregiver and patient characteristics, such as the Spanish version of the SCODI (AD) or the “six item screener” questionnaire [38]. All data collection procedures were managed directly by trained technical staff to ensure consistency and accuracy.

#### 2.2.3. Statistical Analysis

Descriptive statistics were applied to summarize the sample’s sociodemographic characteristics (frequencies and percentages) and the CC-SCODI item and subscale scores (mean, standard deviation, skewness, and kurtosis).

To evaluate the dimensionality of the CC-SCODI, four separate confirmatory factor analyses (CFAs) were conducted, one for each of the four subscales: CC to Self-care Maintenance scale, CC to Self-Care Monitoring scale, CC to Self-Care Management scale, and caregiver self-efficacy in contributing to patient scale. Additionally, an overall model was tested using the four CC-SCODI scales to assess the instrument’s theoretical underpinnings. The factor structure tested in the CFA mirrored that of the SCODI, as both instruments are grounded in the Mid-Range Theory of Chronic Illness Self-Care [27].

Model fit was assessed using the Comparative Fit Index (CFI), Tucker–Lewis Index (TLI), Root Mean Square Error of Approximation (RMSEA), and Standardized Root Mean Square Residual (SRMR). CFI and TLI values between 0.90 and 0.95 indicated a good fit, while values ≥ 0.95 reflected a strong fit [39]. RMSEA values ≤ 0.05 suggested an excellent fit, 0.05–0.08 indicated a good fit, and values ≥0.10 were considered poor [40]. RMSEA 90% confidence intervals ≤0.05–0.08 also indicated a good fit [40]. The close-fit test (*p* > 0.05) was used to evaluate the likelihood of minimal approximation error. SRMR values < 0.08 were indicative of a well-fitting model [39]. The chi-square test was interpreted alongside these indices to provide additional context.

#### 2.2.4. Reliability and Validity Testing

Internal consistency reliability was assessed using Cronbach’s α and McDonald’s omega coefficients. Concurrent validity was evaluated by comparing the scores of each CC-SCODI scale with the corresponding SCODI scale (e.g., CC to Self-Care Maintenance with Patient Self-Care Maintenance). This approach was based on the theoretical premise that caregiver contributions to self-care support patient self-care behaviors.

Pearson’s correlation coefficient (r) was used to assess relationships between variables. Correlations were classified as weak (0.10–0.29), moderate (0.30–0.49), or strong (≥0.50) [41].

#### 2.2.5. Software

All statistical analyses were performed using IBM SPSS-AMOS v24 (IBM Corporation, Armonk, NY, USA) and Jamovi V. 2.6.44 [42].

### 2.3. The CC-SCODI or Caregiver Contribution to Self-Care of Diabetes Inventory

Each participant completed the 40 items of the CC-SCODI in Spanish using a 5-point Likert scale. The CC-SCODI does not provide a single composite score, as it is structured primarily into three subscales that assess the caregiver’s contribution to the three core components of diabetes self-care as described by Riegel’s mid-range theory: CC to Self-Care Maintenance (12 items), CC to Self-Care Monitoring (8 items), and CC to Self-Care Management (9 items). The last subscale assesses caregiver self-efficacy in contributing to patient self-care (11 items), which is caregiver confidence and persistence in supporting the patient’s behaviors investigated in the previous three scales [8,27].

To ensure comparability and adherence to the authors’ guidelines, standardized scores were calculated for each of the four subscales [27,29]. Each subscale score was transformed into a standardized range from 0 to 100, where higher scores indicate a greater caregiver commitment to supporting self-care activities.

The cutoff point for the CC-SCODI subscales was set at 70, consistent with the recommendations of the original tool’s authors and prior studies using similar instruments to evaluate caregiver contributions in other chronic diseases based on the same theoretical framework [30,31,32]. Subscale scores above this threshold indicate adequate caregiver support in the corresponding self-care domain, while scores below 70 suggest potential areas of concern.

The use of standardized scales facilitates the identification of issues and correlations across different aspects of caregiver contributions to self-care. By comparing scores across the four subscales, it becomes possible to pinpoint specific domains where caregiver support may be insufficient and to explore relationships between the various components of self-care contribution.

### 2.4. Aspects of Ethics

The study protocol received approval from the La Rioja Municipal Research Ethics Commission (reference number CEImLAR P.I. 572).

Prior to participating in the study, all individuals provided written informed consent after being thoroughly informed about the study’s objectives, procedures, and their right to withdraw at any time without consequences.

To ensure anonymity and confidentiality, alphanumeric codes were assigned to each participant dyad, and these codes were consistently used throughout the study to identify individuals in the database. No personally identifiable information was linked to the data, ensuring strict compliance with ethical standards. Participation was entirely voluntary, and all participants cooperated willingly. The study protocol adhered to the ethical principles outlined in the Declaration of Helsinki, ensuring respect for participants’ rights, safety, and well-being throughout the research process.

## 3. Results

After meeting the eligibility criteria, a total of 201 caregivers consented to participate in the study. The sociodemographic characteristics of the participants are summarized in Table 1. The majority of caregivers were women (66.77%), married (76.10%), and employed (53.73%). Most participants had children (78.11%), reported having sufficient financial resources to meet their needs (61.19%), did not smoke (74.63%), and did not consume alcohol (74.63%). The primary relationship between the caregiver and the care recipient was that of a spouse or partner (56.22%), with the majority of caregivers cohabitating with the care recipient (85.57%). The mean age of the sample was 54 years.

Table 2 shows the descriptive analysis of the 40 items of the Spanish version of the CC-SCODI. For the sample of 201 caregivers, the descriptive statistics of the CC-SCODI items, including mean, standard deviation (SD), skewness, and kurtosis, were calculated. Not all items followed a normal distribution, as evidenced by skewness and kurtosis indices.

**First Subscale: Caregiver Contribution to Self-Care Maintenance (Items 1–12)**

Among the items in this scale, the question “Keep appointments with your health care provider?” (CC-SCODI10) obtained the highest mean score, indicating strong caregiver support in this area. In contrast, the item “Perform physical exercise for 2 h and 30 min each week? (example: swimming, going to the gym, cycling, walking)” (CC-SCODI2) received the lowest mean score, suggesting less caregiver involvement in promoting regular physical activity.

**Second Subscale: Caregiver Contribution to Self-Care Monitoring (Items 13–20**)

On this scale, the item “Monitor blood sugar regularly?” (CC-SCODI13) achieved the highest mean score, reflecting a high level of caregiver engagement in blood sugar monitoring. Conversely, the item “Monitor blood pressure?” (CC-SCODI15) scored the lowest, indicating a potential gap in caregiver involvement in monitoring blood pressure.

**Third Subscale: Caregiver Contribution to Self-Care Management (Items 21–29)**

The item “If the person you care for finds out that blood sugar is too high or too low, to adjust the insulin dosage in the way your health care provider suggested?” (CC-SCODI29) had the highest mean score, demonstrating strong caregiver support in insulin management. In contrast, the item “When the person you care for has abnormal blood sugar levels, to ask a family member or friend for advice?” (CC-SCODI23) received the lowest score, suggesting limited reliance on external advice in such situations.

**Fourth Subscale: Caregiver Self-Efficacy in Contributing to patient Self-Care (Items 30–40)**

The question “Take medicines in the appropriate way (including insulin if prescribed)?” (CC-SCODI32) obtained the highest score, indicating high caregiver confidence in supporting medication adherence. On the other hand, the item “Prevent high or low blood sugar levels and its symptoms?” (CC-SCODI30) scored the lowest, highlighting a potential area for improvement in caregiver confidence regarding preventive measures for blood sugar control.

**The respondents demonstrated the greatest challenges in the domain of self-care management, with a mean score of 73.17 points (SD = 15.03),** indicating this as the area where caregivers may require additional support or resources. Conversely, participants excelled in maintaining self-care, achieving the highest mean score of 83.94 points (SD = 10.71), reflecting strong caregiver contributions in this domain.

Further details regarding the specific features of each CC-SCODI scale in the Spanish version of the survey are presented in Table 3.

The following are abbreviations: *N*-number of participants; SD-standard deviation; Lo-lowest value (range 0–100); Hi-highest (range 0–100); CC-SCODI-Caregiver Contribution to Self-Care of Diabetes Inventory.

### 3.1. Confirmatory Factor Analysis (CFA) and Model Fit Indices

The parameter estimation method employed was the Robust Weighted Least Squares (WLSMV), a weighted least squares method adjusted for mean and variance. This approach was chosen due to the ordinal nature of the data and differences in variances (B6).

Item #29 (“*If the person you care for finds out that blood sugar is too high or too low, to adjust the insulin dosage in the way your health care provider suggested*”) was excluded from the analysis, as the psychometric evaluation revealed insufficient results to retain it in the model.

The fit indices, including CFI, TLI, SRMR, RMSEA, all yielded satisfactory results for the structure of the Spanish version of the CC-SCODI. These findings support the validity of the original four-component structure of the tool.

Detailed results of the CFA are presented in Table 4.

The Confirmatory Fit Index (CFI) evaluates the degree to which the proposed model improves upon a null model in terms of fit. A CFI score between 0.90 and 0.95 suggests a decent fit, while values ≥ 0.95 indicate an excellent model fit. Higher CFI values represent a better fit to the data.

The Standardized Root Mean Square Residual (SRMR) measures the average discrepancy between observed correlations and model predictions. Lower SRMR values indicate a better fit, with values < 0.08 considered good and values between 0.08 and 0.10 deemed acceptable.

The Root Mean Square Error of Approximation (RMSEA) assesses the model’s fit while accounting for both sample size and model complexity. An RMSEA value < 0.06 indicates a high match, while values between 0.06 and 0.08 are considered acceptable.

For the current model, with 773 degrees of freedom, the chi-square index yielded a score of 1.028. Additionally, a chi-square value of 996 with 696 degrees of freedom was obtained. Given that the chi-square statistics are sensitive to sample size, the chi-square value was divided by the degrees of freedom. Since this ratio is less than 3, the model fit is deemed adequate.

**Figure 1:**
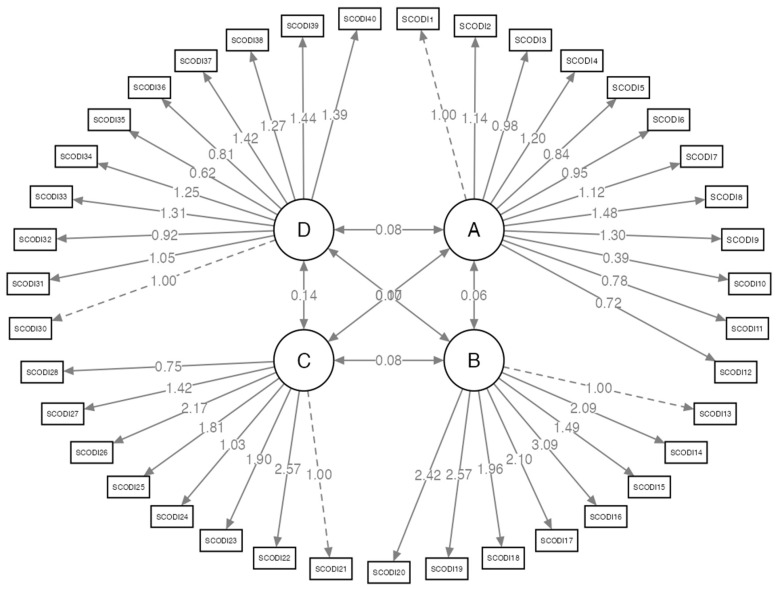
Confirmatory analysis for the CC-SCODI in Spanish. The following are abbreviations: A: Caregiver Contribution to Self-Care Maintenance subscale; B: Caregiver Contribution to Self-Care Monitoring subscale; C: Caregiver Contribution to Self-Care Management subscale; D: caregiver self-efficacy in contributing to patient self-care subscale; SCODI: item from the Caregiver Contribution to Self-Care of Diabetes Inventory.

The confirmatory factor analysis of the Spanish version of the CC-SCODI is illustrated in Figure 1. This figure also displays non-standardized factor loadings. Theoretical constructs, which represent latent variables that cannot be directly measured but are inferred from observable variables, are designated as latent variables A, B, C, and D.

The observed variables, represented by rectangles, correspond to the CC-SCODI items, which serve as indicators for evaluating the latent variables. The arrows pointing from the latent variables to the observed variables signify the influence of the former on the latter. The regression coefficients (factor loadings), displayed alongside the arrows, indicate both the direction and magnitude of the relationships between latent and observed variables.

### 3.2. CC-SCODI Questionnaire Reliability Analysis

The reliability of the Spanish version of the CC-SCODI was assessed using Cronbach’s α and McDonald’s omega coefficients. These measures were employed to evaluate the internal consistency of the items.

In the sample of 201 caregivers, the following Cronbach’s α values were observed for the four subscales of the CC-SCODI: self-care maintenance: 0.725; self-care monitoring: 0.728; self-care management: 0.729; self-efficacy in contributing to patient self-care: 0.921 (Table 5).

Similarly, the McDonald’s omega coefficients for these subscales were as follows: self-care maintenance: 0.767; self-care monitoring: 0.753; self-care management: 0.771; self-efficacy in contributing to patient self-care: 0.922 (Table 5).

These results indicate sufficient reliability for all subscales.

Furthermore, as shown in Table 6, the removal of individual items from the scales did not significantly improve the dependability of the instrument, suggesting that all items contribute meaningfully to the overall reliability.

**Figure 2:**
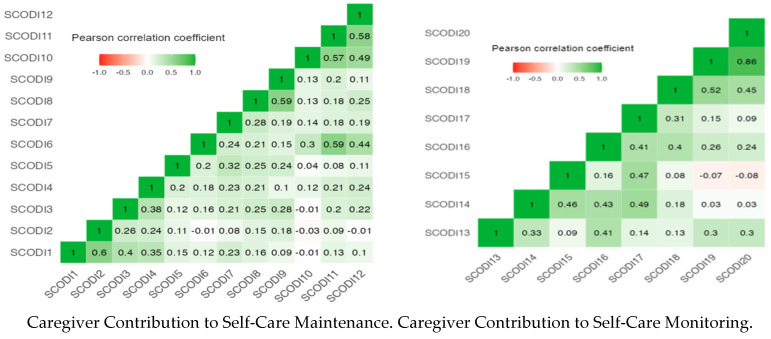

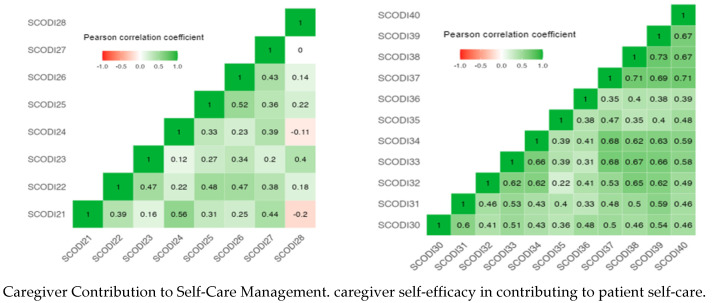
Pearson correlation coefficient heat map of the CC-SCODI scales.

Finally, the relationships among the items within the four scales were found to be acceptable, as illustrated in Figure 2, which highlights the associations among the items.

SCODI is the abbreviation for the item from the Caregiver Contribution to Self-Care of Diabetes Inventory.

### 3.3. Concurrent Validity of the CC-SCODI

Pearson’s r was 0.659 (*p* < 0.001) for the Caregiver Contribution to Self-Care Maintenance scale and the patient Self-Care Maintenance scale; 0.670 (*p* < 0.001) for the Caregiver Contribution to Self-Care Monitoring scale and the patient Self-Care Monitoring scale; 0.809 (*p* < 0.001) for the Caregiver Contribution to Self-Care Management scale and the patient Self-Care Management scale; and 0.627 (*p* < 0.001) for the caregiver self-efficacy in contributing to patient self-care scale and the patient self-care self-efficacy scale.

## 4. Discussion

In order to create a valid and reliable Spanish version of the CC-SCODI, the original instrument required translation and cultural adaptation. This study validated the psychometric properties of the adapted tool, which is grounded in Riegel’s mid-range theory of chronic illness [8]. This theoretical framework has significantly influenced the development of psychometrically robust, disease-specific instruments to evaluate self-care and caregiver contributions (CCs) to self-care. Self-care is conceptualized in three dimensions: management, vigilance, and maintenance, with self-efficacy playing a critical role in each. The original CC-SCODI questionnaire employs four scales to assess the contributions of caregivers to self-care [29], and our results demonstrate that the CC-SCODI is a reliable and effective tool for measuring caregiver contributions, as evidenced by the responses of 201 caregivers of individuals with T1DM and T2DM.

The psychometric validation of the CC-SCODI revealed that the SRMR, RMSEA, CFI, and TLI fit indices all exhibited appropriate values, supporting the structural validity of the instrument. However, Item #29, which assesses the caregiver’s role in adjusting insulin dosage based on blood sugar levels, raised concerns regarding its validity within the Caregiver Contribution to Self-Care Maintenance scale. This item is conditional, as it applies only to caregivers of individuals who use insulin. Among the 157 caregivers who answered this question, 73 cared for individuals with T1DM and 84 for individuals with T2DM. No significant differences were observed between the two groups, with 93.8% of respondents selecting “always” (5 points), 5.7% selecting “often” (4 points), and only 0.6% selecting “sometimes,” while no responses were recorded for lower scores. This lack of variability suggests that the item does not effectively differentiate between respondents, which undermines its reliability. Consequently, Item #29 was excluded from subsequent analyses but retained in the instrument for further psychometric evaluation in future studies to determine whether this issue reflects a sample-specific bias, a cultural characteristic of Spanish caregivers, or a broader trend across different populations. Similarly, in the validation of the original CC-SCODI, Item #29 was also excluded in the confirmatory factor analysis [29].

The mean scores of our sample, for the standardized scores obtained in the four scales of the CC-SCODI, showed adequate behaviors in all of them. However, in line with those of previous international studies, both for patients and caregivers, the highest scores were observed in the maintenance scales (83.94 in the Spanish CC-SCODI; 56.17 in the original CC-SCODI; 78.24 in the Spanish SCODI) and self-efficacy (82.63, 68.69, and 79.56, respectively), followed by the monitoring scale (77.36, 51.81, and 71.81, respectively), with the lowest scores for management (73.17, 49.47, and 62.85, respectively) [23,29]. Although they should be followed up in further studies, it highlights the need to intensify efforts to increase knowledge of self-care management behaviors, both consultative and autonomous, in people with DM and their caregivers. Specific results depend on the sample, but trends may provide relevant information.

The original version of the CC-SCODI was validated with a sample of 251 informal caregivers of people with T2DM, obtaining reliability results with the McDonald’s omega coefficient of 0.87 for Caregiver Contribution to Self-Care Maintenance, 0.78 for Caregiver Contribution to Self-Care Monitoring, 0.89 for Caregiver Contribution to Self-Care Management, and 0.88 for caregiver self-efficacy in contributing to patient self-care [29]. For the Spanish version of the CC-SCODI, 201 informal caregivers of people with both T1DM and T2DM were selected, obtaining equally adequate reliability results. The literature has previously demonstrated [28] the validity of the patient version of the SCODI for both groups, and in this study, no significant differences were observed between them.

Concurrent validity between the four CC-SCODI subscales and the four SCODI subscales confirmed the hypothesis that when caregivers scored high or low on caregiver self-efficacy to contribute to patient self-care, patients scored high or low, respectively, on their self-care, demonstrating the high influence of informal caregivers, and thus their high clinical relevance.

Instruments like the CC-SCODI are crucial in research and clinical practice, as they provide rapid, reliable insights into the behaviors, strengths, and limitations of individuals with DM in terms of self-care. Identifying individuals at risk of suboptimal self-care outcomes is greatly facilitated by the availability of valid tools like the CC-SCODI. Effective DM management requires a comprehensive, interdisciplinary approach that involves patients and their families while addressing behavioral, emotional, psychological, and educational factors [43]. Regular evaluation of self-care practices, with the assistance of caregivers, is essential for identifying deficiencies and implementing tailored interventions [44]. The CC-SCODI fulfills this need by offering a structured framework for assessing caregiver contributions to self-care, bridging a significant gap in the Spanish literature on caregiver roles in DM management.

Although caregiver contributions to self-care have been recognized as critical for improving outcomes in chronic illnesses, including DM, the Spanish version of the CC-SCODI had not been previously validated, leaving a notable void in the research. Existing studies have primarily focused on outcomes such as glycemic control or therapy adherence without exploring the specific dimensions of self-care or caregiver behaviors. For example, family empowerment [45] and caregiver strain [46] have been shown to influence self-care outcomes, while caregivers have been found to improve therapy adherence in chronic conditions like DM [47]. Family support has also been identified as a key factor in facilitating the use of health information technologies by patients [48]. Programs that combine telemonitoring and caregiver engagement have demonstrated significant improvements in medication adherence, glucose monitoring, and foot inspections among patients with type 2 diabetes mellitus [49]. These findings underscore the potential utility of the CC-SCODI in evaluating and enhancing caregiver contributions to self-care.

The increasing adoption of remote monitoring technologies in healthcare, particularly during the COVID-19 pandemic, has further highlighted the importance of caregiver involvement [50]. Remote monitoring facilitates real-time evaluation of patient outcomes, reduces the frequency of hospital visits, and enables care delivery to underserved populations [51,52]. Caregivers play a critical role in bridging knowledge and skill gaps, empowering patients to effectively use these technologies [22]. Research has shown that family empowerment strategies can significantly enhance self-care in individuals with diabetes [15], while a lack of social support and depressive symptoms are associated with poor outcomes [53]. Understanding how interventions and educational programs impact caregivers and patients is essential for designing personalized strategies to improve self-care practices.

Caregivers can have both positive and negative effects on the self-care efforts and health outcomes of individuals with diabetes mellitus. Programs that emphasize the role of informal caregivers in enhancing self-care have shown promising results, particularly when tailored to the specific needs of patients and caregivers [7,54]. For example, patients with DM1 have demonstrated better outcomes when family-centered programs are personalized compared to more generalized approaches [7]. The CC-SCODI provides valuable insights into caregiver behaviors and their influence on various aspects of self-care. When combined with the SCODI, the CC-SCODI enables healthcare providers to identify at-risk dyads and implement targeted interventions, such as adjusting the frequency of medical consultations, developing health education programs, and fostering confidence and motivation.

The perspective of individuals with DM and their caregivers underscores the importance of individualized, contextualized, and self-paced training and counseling programs [55]. Grouping patients and caregivers with similar characteristics has also been shown to foster a sense of community and belonging, which can enhance program outcomes [56]. However, these approaches may strain limited healthcare resources and workloads. The CC-SCODI addresses this challenge by facilitating the categorization and organization of caregivers based on their unique traits, enabling more efficient allocation of resources.

Despite the benefits of caregiver contributions to self-care, informal caregivers often experience stress, burnout, and physical or psychological issues, such as headaches, fatigue, and depression, particularly when they lack the necessary knowledge or skills [57]. Identifying ineffective caregivers is crucial, as it not only prevents burnout but also ensures better support for patients. The CC-SCODI fills this gap by providing a reliable tool for assessing caregiver effectiveness and identifying areas for intervention.

Research has also highlighted the value of dyadic approaches to self-care, which consider the dynamic interactions between patients and caregivers. Dyadic self-care behaviors, characterized by the exchange of emotions, thoughts, and actions, have been shown to influence self-care practices [58]. For example, while self-care monitoring did not benefit from dyadic interventions, self-care management and maintenance did [59]. The combination of the CC-SCODI and SCODI facilitates dyadic studies in diabetes care, providing a comprehensive framework for evaluating self-care processes and designing effective interventions. Frequent assessment of these factors can help identify dyads at risk of poor self-care practices, enabling early intervention and improved outcomes.

In conclusion, the CC-SCODI is a valid and reliable tool for assessing caregiver contributions to self-care in individuals with DM. Its application in research and clinical practice can enhance our understanding of caregiver roles, support the development of personalized interventions, and ultimately improve the quality of life for patients and their caregivers. Future studies should focus on further psychometric evaluation of the CC-SCODI, particularly conditional items like Item #29, and explore its utility in diverse cultural and clinical contexts.

## 5. Limitations and Implications for Nursing

The CC-SCODI is part of a group of questionnaires derived from Riegel’s middle-range theory, designed to measure caregivers’ contributions to self-care in various chronic diseases. This tool is particularly relevant for nursing, as it captures the three main dimensions of self-care—maintenance, monitoring, and management—along with self-efficacy, which is critical for effective caregiving. The outcomes of the CC-SCODI are influenced by numerous sociodemographic and clinical variables, such as depression, anxiety, socioeconomic status, mutuality, and health literacy, areas where nursing interventions play a crucial role in mitigating negative impacts and enhancing self-care behaviors.

Despite its strengths, several limitations must be acknowledged to ensure its effective application in nursing practice and research. First, the results should be interpreted with caution when applied to diverse cultures and nations, as the study used a convenience sample collected in a specific geographic location. This limits the generalizability of the findings to broader populations. Additionally, the cross-sectional design of the study restricts its ability to explore causal relationships or changes over time; future longitudinal research would provide deeper insights into the dynamic interplay between caregivers and patients.

Another limitation is the sampling method, which may introduce Berkson bias, as all caregivers included in the study were present at the hospital due to the hospitalization of the person they were caring for. However, this bias is unlikely to significantly affect the results, as hospital admissions were not directly related to the effects of diabetes mellitus (DM). Additionally, patients with significant cognitive impairments were excluded from the study to ensure accurate assessment of caregiver contributions, which may limit the tool’s applicability to caregiver–patient dyads where cognitive impairment is a factor.

On the other hand, it is important to recognize that self-care is significantly influenced by the caregiver’s behaviors, perceptions of the illness, and cultural factors.

For nursing, the CC-SCODI offers a validated starting point to measure and understand the influence of various factors on caregiver contributions to self-care. By addressing the limitations through future research—such as expanding cultural applicability, conducting longitudinal studies, and including diverse caregiver–patient dyads—nurses can further refine this tool to optimize care strategies. This highlights the indispensable role of nursing in interpreting, applying, and advancing the use of such tools to improve patient and caregiver outcomes in chronic disease management.

## 6. Conclusions

Our study demonstrated that the Spanish version of the CC-SCODI exhibits strong psychometric properties in terms of validity and reliability. Consequently, it is a suitable instrument for assessing caregiver involvement in the self-care of individuals with diabetes, making it a valuable tool for nursing clinical practice and research applications.

## Figures and Tables

**Table 1:** Caregivers’ sociodemographic characteristics (N = 201).

Variable	Mean (Range)	SD
**Age (years)**	54.12 (22–88)	12.99
**Sociodemographic Factors**	**n**	**%**
**Gender**		
**Male**	67	33.33
**Female**	134	66.77
**Total**	201	100
**Marital Status**		
**Single/Unmarried**	30	14.90
**Married**	153	76.10
**Separated/Divorced**	15	7.50
**Widower**	3	1.50
**Total**	201	100
**Education Level**		
**Elementary**	46	22.88
**Secondary school**	40	19.90
**Diploma**	52	25.87
**Bachelor’s degree**	13	6.47
**University**	50	24.88
**Total**	201	100
**Currently Occupation**		
**Employee**	108	53.73
**Self-Employed**	43	21.39
**Retired**	50	24.88
**Total**	201	100
**Number of people living with the caregiver**		
**0**	5	2.49
**1**	74	36.82
**2**	48	23.89
**3**	57	28.35
**>3**	17	8.45
**Total**	201	100
**Children**		
**0**	44	21.89
**1**	47	23.38
**2**	88	43.78
**3**	19	9.46
**>3**	3	1.49
**Total**	201	100
**Perceived Income Adequacy**		
**More than needed**	60	29.85
**Enough for living**	123	61.19
**Less than needed**	18	8.96
**Total**	201	100
**Smoker**		
**Yes**	51	25.37
**No**	150	74.63
**Total**	201	100
**Drink Alcohol**		
**Yes**	51	25.37
**No**	150	74.63
**Total**	201	100
**Relationship with Care Recipient**		
**Spouse/partner**	113	56.22
**Son/daughter**	40	19.90
**Parents**	41	20.40
**Others**	7	3.48
**Total**	201	100
**Cohabitation with Care Recipient**		
**Yes**	172	85.57
**No**	29	14.43
**Total**	201	100

**Table 2:** Descriptive of the items that make up CC-SCODI.

Item	Mean	SD	Skewness	Kurtosis
Subscale A: Caregiver contribution to self-care maintenance.				
CC-SCODI1	4.21	0.96	−0.95	−0.26
CC-SCODI2	3.15	1.46	−0.16	−1.31
CC-SCODI3	4.31	0.76	−0.87	0.24
CC-SCODI4	4.00	0.88	−0.59	−0.36
CC-SCODI5	4.50	0.91	−2.17	4.70
CC-SCODI6	4.77	0.56	−3.22	13.38
CC-SCODI7	4.57	0.93	−2.43	5.60
CC-SCODI8	4.10	0.99	−0.72	−0.56
CC-SCODI9	3.92	1.06	−0.66	−0.29
CC-SCODI10	4.96	0.30	−8.04	67.60
CC-SCODI11	4.91	0.39	−5.43	33.50
CC-SCODI12	4.89	0.38	−4.06	20.79
Subscale B: Caregiver contribution to self-care monitoring.				
CC-SCODI13	4.77	0.57	−2.88	8.75
CC-SCODI14	4.00	1.07	−0.89	0.15
CC-SCODI15	3.07	1.34	−0.11	−1.03
CC-SCODI16	4.07	1.29	−1.19	0.24
CC-SCODI17	3.68	1.11	−0.63	−0.12
CC-SCODI18	4.68	0.76	−3.26	12.22
CC-SCODI19	4.01	1.23	−1.51	2.12
CC-SCODI20	4.02	1.28	−1.51	1.98
Subscale C: Caregiver contribution to self-care management.				
CC-SCODI21	4.71	0.59	−2.21	4.99
CC-SCODI22	3.49	1.26	−0.45	−0.81
CC-SCODI23	2.88	1.40	0.03	−1.24
CC-SCODI24	4.70	0.65	−2.73	8.83
CC-SCODI25	4.32	0.91	−1.52	2.18
CC-SCODI26	3.57	1.13	−0.73	0.11
CC-SCODI27	4.62	0.78	−2.47	6.45
CC-SCODI28	2.39	1.49	0.61	−1.09
CC-SCODI29	4.93	0.28	−4.25	19.28
Subscale D: Caregiver self-efficacy in contributing to patient self-care.				
CC-SCODI30	3.82	0.83	−0.17	−0.39
CC-SCODI31	4.17	0.85	−0.93	0.84
CC-SCODI32	4.67	0.67	−2.79	10.17
CC-SCODI33	4.30	0.85	−1.36	1.99
CC-SCODI34	4.34	0.85	−1.85	1.90
CC-SCODI35	4.58	0.64	−1.26	0.43
CC-SCODI36	4.19	0.83	−0.84	0.38
CC-SCODI37	4.31	0.89	−1.27	1.27
CC-SCODI38	4.43	0.79	−1.67	3.39
CC-SCODI39	4.36	0.89	−1.62	2.77
CC-SCODI40	4.19	0.92	−1.12	0.83

**Table 3:** CC-SCODI scale characteristics.

CC-SCODI	N	Mean	SD	Median	Lo	Hi	Range
Caregiver contribution to self-care maintenance items 1–12	201	83.94	10.71	85.41	50	100	50
Caregiver contribution to self-care monitoring items 13–20	201	77.36	15.34	79.41	29.41	100	70.59
Caregiver contribution to self-care management items 21–29	201	73.17	15.03	75	18.75	100	81.25
Caregiver self-efficacy in contributing to patient self-care items 30–40	201	82.63	15.37	84.09	25	100	75

**Table 4:** The tested SCODI models’ CFA fit indices.

**USER MODEL VERSUS BASELINE MODEL**					
					**Value**
Comparative Fit Index (CFI)					0.954
Tucker–Lewis Index (TLI)					0.951
Bentler–Bonett Non-normed Fit Index (NNFI)					0.951
Bentler–Bonett Normed Fit Index (NFI)					0.864
Parsimony Normed Fit Index (PNFI)					0.811
Bollen’s Relative Fit Index (RFI)					0.855
Bollen’s Incremental Fit Index (IFI)					0.955
Relative No centrality Index (RNI)					0.954
**FIT INDICES**					
			**95% Confidence Intervals**		
**Type**	**SRMR**	**RMSEA**	**Lower**	**Upper**	**RMSEA value**
Classical	0.104	0.046	0.040	0.053	0.817
Robust	0.104				
Scaled	0.104	0.042	0.035	0.049	0.978

Note: The RMSEA (Root Mean Square Error of Approximation) has an associated *p*-value, known as the RMSEA *p*-value, which evaluates the null hypothesis that the model has a perfect fit in the population (RMSEA = 0). For the classical RMSEA in this analysis, the *p*-value is 0.817, indicating that there is no significant evidence of poor fit. For the scaled RMSEA, the *p*-value is 0.978, which also indicates a good fit.

**Table 5:** Reliability analysis of the CC-SCODI scales.

CC-SCODI	Cronbach’s α	McDonald’s Omega
Caregiver Contribution to Self-Care Maintenance	0.725	0.767
Caregiver Contribution to Self-Care Monitoring	0.728	0.753
Caregiver Contribution to Self-Care Management	0.729	0.771
Caregiver self-efficacy in contributing to patient self-care	0.921	0.922

**Table 6:** Reliability analysis of the CC-SCODI items.

CC-SCODI Maintenance			CC-SCODI Monitoring			CC-SCODI Management			CC-SCODI Self-Efficacy		
Item	If Item Deleted		Item	If Item Deleted		Item	If Item Deleted		Item	If Item Deleted	
	C’s α	McD’s ω		C’s α	McD’s ω		C’s α	McD’s ω		C’s α	McD’s ω
1	0.688	0.755	13	0.713	0.738	21	0.718	0.750	30	0.916	0.918
2	0.727	0.765	14	0.694	0.739	22	0.656	0.721	31	0.916	0.918
3	0.695	0.751	15	0.744	0.763	23	0.682	0.753	32	0.914	0.916
4	0.699	0.751	16	0.679	0.721	24	0.723	0.759	33	0.910	0.912
5	0.712	0.762	17	0.683	0.734	25	0.676	0.726	34	0.911	0.913
6	0.713	0.745	18	0.693	0.717	26	0.674	0.730	35	0.921	0.923
7	0.705	0.754	19	0.691	0.723	27	0.701	0.738	36	0.922	0.924
8	0.690	0.747	20	0.702	0.730	28	0.766	0.792	37	0.908	0.910
9	0.701	0.754							38	0.909	0.911
10	0.725	0.759							39	0.907	0.910
11	0.713	0.736							40	0.911	0.913
12	0.716	0.742									

Note: C’s α = Cronbach’s α; McD’s ω = McDonald’s omega.

## Data Availability

Data are available upon request from the first author due to legal restrictions.

## Public Involvement Statement

No public involvement in any aspect of this research.

## Guidelines and Standards Statement

This manuscript was drafted against the STROBE guideline.
